# Timely surveillance and temporal calibration of disease response against human infectious diseases

**DOI:** 10.1371/journal.pone.0258332

**Published:** 2021-10-18

**Authors:** Kamran Najeebullah, Jessica Liebig, Jonathan Darbro, Raja Jurdak, Dean Paini

**Affiliations:** 1 Data61, Commonwealth Scientific and Industrial Research Organisation, Dutton Park, Australia; 2 Health & Biosecurity, Commonwealth Scientific and Industrial Research Organisation, Dutton Park, Australia; 3 Metro North Public Health Unit, Queensland Health, Brisbane, Queensland, Australia; 4 Department of Computer Science, Queensland University of Technology, Brisbane, Australia; Swedish National Veterinary Institute, SWEDEN

## Abstract

**Background:**

Disease surveillance and response are critical components of epidemic preparedness. The disease response, in most cases, is a set of reactive measures that follow the outcomes of the disease surveillance. Hence, timely surveillance is a prerequisite for an effective response.

**Methodology/principal findings:**

We apply epidemiological soundness criteria in combination with the Latent Influence Point Process and time-to-event models to construct a disease spread network. The network implicitly quantifies the fertility (whether a case leads to secondary cases) and reproduction (number of secondary cases per infectious case) of the cases as well as the size and generations (of the infection chain) of the outbreaks. We test our approach by applying it to historic dengue case data from Australia. Using the data, we empirically confirm that high morbidity relates positively with delay in disease response. Moreover, we identify what constitutes timely surveillance by applying various thresholds of disease response delay to the network and report their impact on case fertility, reproduction, number of generations and ultimately, outbreak size. We observe that enforcing a response delay threshold of 5 days leads to a large average reduction across all parameters (occurrence 87%, reproduction 83%, outbreak size 80% and outbreak generations 47%), whereas extending the threshold to 10 days, in comparison, significantly limits the effectiveness of the response actions. Lastly, we identify the components of the disease surveillance system that can be calibrated to achieve the identified thresholds.

**Conclusion:**

We identify practically achievable, timely surveillance thresholds (on temporal scale) that lead to an effective response and identify how they can be satisfied. Our approach can be utilized to provide guidelines on spatially and demographically targeted resource allocation for public awareness campaigns as well as to improve diagnostic abilities and turn-around times for the doctors and laboratories involved.

## Introduction

Sporadic outbursts of emerging and re-emerging infectious diseases are on the rise, carrying catastrophic consequences for health and livelihoods of people in poor and wealthy parts of the world alike [1–3]. Fueled by the effects of globalization, the growing mobility of human populations and constant urbanization, epidemics can reach pandemic scale in a matter of days [4]. A warming and unstable climate, increasing population density and frequent interactions between humans and wild animals are likely to further amplify the risk of emerging diseases [3, 4], posing an imminent threat to global health security.

Epidemic preparedness is an indispensable element of health security in mitigating the threat of infectious diseases. Timely surveillance and effective response capabilities are pivotal for epidemic preparedness [5]. A disease surveillance system is a tool to detect, confirm and report the occurrence of a disease in a population. Disease response, on the other hand, is a set of reactive (to the outcome of surveillance) actions to reduce morbidity and consequent mortality [6]. Disease response is not an exact science and the actions to control an infectious disease may differ due to variations in factors including seasonality, climate, individual susceptibility, community values, and characteristics of the pathogen itself. Regardless of the context, experts agree that minimizing response time is critical for epidemic control [7–9]. The disease response, in most cases, is a set of reactive measures that follow the outcomes of the disease surveillance. Thus implying, timely surveillance is a prerequisite for an effective response.

In this paper we introduce a methodology that quantifies the timeliness of a disease surveillance system. To this end, we construct a *disease spread network* to identify disease pathways by defining parent-child relationships between the individual cases. We apply *epidemiological soundness* criteria in combination with the *Latent Influence Point Process* (LIPP) [10] and *time-to-event* [11] models to estimate these relations by utilizing the disease occurrence, meteorological and human population mobility data. Unlike prior studies where the timeliness of the disease surveillance is measured by comparing the average notification delay and its components to predefined, standardized and/or disease specific timeframes [12–18], our methodology quantifies the timeliness by measuring the case fertility (whether a case leads to secondary cases) and reproduction (number of secondary cases per fertile case) as well as the outbreak size and generations (of the infection chain). While the traditional approach is important to identify the overall performance gaps, our approach goes a step further by measuring the impact of these gaps on the occurrence of the disease. The construction of the disease spread network allows us to empirically confirm that high morbidity relates positively with delay in disease response. Furthermore, we test varying thresholds for what may represent timely surveillance and gauge their impact on various parameters associated with the disease occurrence. Lastly, we conduct a spatio-temporal analysis of the delays involved at the various stages of the disease surveillance, identify bottlenecks, and provide insights on how they may be prevented. Our results can be utilized to provide guidelines on spatially and demographically targeted resource allocation for public awareness campaigns as well as to improve diagnostic abilities and turn-around times for the doctors and laboratories involved. The timely surveillance thresholds obtained in this study can be utilized to define a case ranking system that can help prioritize testing of the cases that are more likely to lead to larger outbreaks.

## Materials and methods

This study was approved by the CSIRO Social Science Human Research Ethics Committee (Ethics Clearance 065/19) and by the Health Innovation, Investment and Research Office (HIIRO) under section 284 of the Public Health Act 2005 (grant number: RD007950). All data were analysed anonymously and individuals cannot be identified.

### Notions

**Fig 1:**
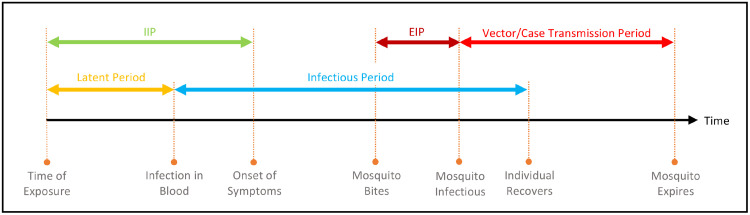
Dengue infection timeline. The blue, yellow and green arrows depict the dengue infection timeline in a human, while the two red arrows depict the same for a mosquito. A human undergoes a latent, intrinsic incubation (IIP) and infectious period indicating the time span during which it is infected: but not infectious, but not showing symptoms, and infectious, respectively. A mosquito undergoes an extrinsic incubation (EIP) and transmission period during which it is infected: but not infectious, and infectious, respectively. A case transmission period is an aggregation of all vector transmission periods associated with the case that starts with the earliest concluding EIP and ends with the death of the last infected mosquito.

We choose dengue as the infectious disease to implement our methodology. Our choice is motivated by the availability and the length of the temporal window of the dataset. Dengue is transmitted when an infectious female vector, primarily *Aedes aegypti*, bites a susceptible person, formally known as *time of exposure*. Symptoms of the infection start to appear within 3 to 10 days [11], a period of time known as the *intrinsic incubation period* (IIP). The *latent period* (LP) ends up to 2 days prior to the onset of symptoms [19] and can only be observed through a lab test. A person is capable of providing an infectious blood meal after the end of the latent period, and retains this capability for up to 7 days after the symptom onset [20]. This period is referred to as the *infectious period*. A susceptible mosquito undergoes an *extrinsic incubation period* (EIP) after having an infectious blood meal. The duration of the EIP is influenced by a range of factors including the ambient temperature, and has been reported to be as short as 3 days [11]. In unfavourable conditions (e.g., when temperatures drop below 20°Celsius), the EIP is estimated to be longer than a typical mosquito’s lifetime [11] (i.e., the mosquito dies before the end of its incubation period). In order to facilitate our analysis, we introduce the notions of *transmission period*. We know that, once infected, a mosquito remains infectious for life. We also know that it can take multiple blood meals over its lifetime [21]. Accordingly, we define the period that begins at the end of the EIP and persists until the death of the infectious mosquito as the *vector transmission period*. Since an infectious person may infect multiple mosquitoes, the transmission period for a particular dengue case (called the *case transmission period*) is an aggregation of the vector transmission periods. It starts with the earliest concluding EIP and ends with the death of the last infected mosquito (S1 Text). See Fig 1 for a visual depiction of the dengue infection timeline. For the sake of brevity, we some time refer to the case transmission period simply as the transmission period.

We evaluate the timeliness of surveillance through its impact on disease incidence using four epidemiological indicators: case fertility, reproduction, outbreak size and generation. We define a case to be fertile if it leads to a secondary case (i.e., it is the parent of at least one case that we call its child). The reproduction of a case indicates the number of child cases it has caused. An outbreak is a set of cases connected by the chain of infections (through parent child relations). The size of an outbreak denotes the number of cases in an outbreak. Let the first case of an outbreak be called root. An outbreak generation is a set of cases that are at the same distance (by number of parent child relations) from root. The number of generations in an outbreak are called the depth of the outbreak.

### Dengue in Australia

To evaluate our methodology, we take the reported occurrence of dengue in Australia, between 2002 and 2018, as a case study. Dengue is not endemic in Australia. However, regular annual local outbreaks are reported, triggered by infected travellers arriving from endemic countries [22]. Clearly, the transmission of dengue is subject to the presence of the vector. In Australia, the vector presence is limited to parts of the state of Queensland. The primary vector, *Aedes aegypti*, is prevalent in northern coastal communities and has also been recorded in several areas of central and southern Queensland [22, 23]. The presence of *Aedes albopictus*, a secondary vector capable of transmitting dengue infection, has also been recorded in the northern parts (Torres Strait Islands) of the state [24]. Queensland Health has a passive surveillance system in place for identifying dengue cases. The system relies on general practitioners, emergency departments and laboratories notifying Queensland Health of suspected and laboratory confirmed cases. The treating doctors are advised to report a case upon clinical suspicion under the provision of the Public Health Act 2005 [25]. Since dengue is not endemic in Australia, particular attention is paid to people who exhibit dengue symptoms and have recently travelled to an endemic country. Queensland Health defines a dengue outbreak as at least one locally acquired case [22]. All laboratory-confirmed and suspected cases are notified to the local Public Health Unit (PHU). Upon notification the PHU communicates with the patient as well as the treating doctor and laboratory to collect case details. This includes the spatio-temporal aspects of the case(see Data Collection section for details). Vector elimination is the first line of defence for Queensland Health to prevent the wider spread of dengue. According to the Queensland Dengue Management Plan [22], when a dengue case is notified it is referred to a medical entomologist by the concerned PHU. If the reported case is in a region with known vector presence, the medical entomologist instigates appropriate vector control measures. The measures consist of indoor residual spraying and treating water containers using insect growth regulators or pesticides at the addresses identified through the travel and contact history of the patient. The aim is to eradicate the virus by eliminating the vector within a radius of about 200 metres (the estimated distance a vector can travel over its lifetime) [26]. Queensland Health works collaboratively with local governments and dedicated Dengue Action Response Teams to manage the efforts.

Since vector control in Queensland is a reactive exercise, it is more effective when conducted closer in time to the start of the infectious period. Theoretically, if conducted within the EIP (the beginning of EIP and infectious period can coincide as a susceptible mosquito may bite the infectious person on their first day of infectiousness), the efforts can potentially prevent any subsequent local transmission. However, prompt reporting of a case may not always be possible due to various factors, including awareness and willingness of a patient to seek medical advice, training and diagnostic ability of the treating doctor, capability and capacity of the testing laboratory and timeliness and complexity of the disease surveillance mechanism. We call the time elapsed (in days) since a case has become infectious until it has been notified to Queensland Health the *notification delay*.

### Data collection

Dengue is a notifiable disease in Australia under the Public Health Act 2005 and laboratory confirmed cases are required to be notified to health departments within each state and territory [25]. Since the impact of disease response can only be measured in the regions with local transmission, we limit the scope of our analysis to these regions. Data on the occurrence of dengue was obtained from the Communicable Disease Branch (CDB) of Queensland Health for the period of October 2001 to September 2018. During this period 5,272 cases were reported, of which 2,808 (53%) were acquired locally. The data records cases on an individual level and covers their spatial, temporaland epidemiological aspects. Spatial information includes the place of residence and the place of acquisition on locality/suburb and postcode levels, respectively. For cases acquired overseas the highest resolution for the place of acquisition is the country. Temporal information consists of the date of symptom onset, specimen collection, diagnosis and notification. The epidemiological data contains information about the serotype of the infection.

Traditionally, temporal analysis of the dengue cases is performed with an observation window set for each calendar year, where cases are assigned to a window with respect to their date of symptom onset. Since the majority of locally acquired dengue cases in Queensland occur between October and May, we move the observation window to align it with this seasonal fluctuation. For each year, our observation window starts at the beginning of September of the previous year and concludes at the end of August of the current year. This shift allows us to incorporate the outbreaks that span across calendar years (see Table 1 for a year-wise breakdown of the occurrence). For the ease of analysis, spatial statistics are aggregated by Statistical Area Level 4 (SA4). The SA4s are sub-State regions defined by the Australian Statistical Geography Standard (ASGS) with a population range between 100,000 and 500,000 individuals. Queensland has 19 SA4 regions in total, of which 7, namely Cairns, Darling Downs, Fitzroy, Mackay, Queensland-Outback, Townsville and Wide Bay, have recorded the presence of the vector.

**Table 1:** Statistics on dengue occurrence and dynamics of its spread in Queensland, Australia. The Fertility, Parent and Outbreak columns present the average (value outside the parentheses) and confidence interval values that were computed by applying the Monte Carlo’s method to the construction of the disease spread network. A case is fertile if it has resulted in at least one secondary case and the reproduction indicates the average number of secondary cases per fertile case. The parent of a locally acquired case is unknown if no suitable parent case was found during the construction of the disease spread network and known otherwise.An outbreak is a set of cases connected by the chain of infections (through parent child relations). The size of an outbreak denotes the number of cases in an outbreak. Let the first case of an outbreak be called root. An outbreak generation is a set of cases that are at the same distance (by number of parent child relations) from root. The number of generations in an outbreak are called the depth of the outbreak.

Year	Cases	Acquisition		Fertility (95% CI)		Parent (95% CI)		Outbreaks (95% CI)	
		Local	Overseas	Fertile	Reproduction	Known	Unknown	Size	Depth
2002	70	27	43	2 (2.0, 2.0)	1 (1.0, 1.0)	2 (2.0, 2.0)	25 (25.0, 25.0)	2 (2.0, 2.0)	2 (2.0, 2.0)
2003	541	485	56	75.3 (74.8, 75.9)	6.1 (5.9, 6.4)	463.7 (463.6, 463.8)	21.3 (21.2, 21.4)	28.0 (27.8, 28.2)	3.5 (3.5, 3.5)
2004	455	412	43	77.9 (77.0, 78.8)	4.8 (4.7, 5.0)	377.7 (377.5, 377.9)	34.2 (34.0, 34.4)	27.2 (26.9, 27.5)	3.6 (3.6, 3.6)
2005	105	73	32	18.6 (18.2, 19.0)	2.9 (2.8, 3.0)	54.1 (54.0, 54.2)	18.9 (18.8, 19.0)	12.5 (12.4, 12.7)	3.8 (3.8, 3.8)
2006	73	38	35	12.6 (12.4, 12.8)	1.7 (1.6, 1.7)	21.1 (21.0, 21.2)	16.9 (16.8, 17.0)	4.9 (4.8, 5.0)	3.1 (3.1, 3.1)
2007	113	46	67	15.4 (15.2, 15.7)	2.7 (2.6, 2.8)	42.2 (42.2, 42.3)	3.7 (3.7, 3.8)	31.1 (30.2, 32.0)	6.4 (6.3, 6.4)
2008	102	22	80	4.6 (4.4, 4.8)	1.3 (1.2, 1.3)	5.9 (5.9, 6.0)	16.0 (16.0, 16.1)	2.7 (2.7, 2.8)	2.3 (2.3, 2.3)
2009	1110	998	112	153.6 (152.4, 154.9)	6.2 (6.0, 6.3)	946.8 (946.5, 947.1)	51.2 (50.9, 51.5)	63.1 (62.4, 63.7)	4.3 (4.2, 4.3)
2010	224	57	167	8.1 (7.9, 8.3)	2.4 (2.3, 2.5)	19.5 (19.4, 19.7)	37.4 (37.3, 37.6)	3.8 (3.8, 3.8)	2.2 (2.2, 2.2)
2011	249	103	146	24.6 (24.3, 24.9)	2.8 (2.7, 2.9)	68.4 (68.3, 68.6)	34.5 (34.4, 34.7)	7.9 (7.8, 8.0)	2.5 (2.5, 2.5)
2012	225	16	209	3.8 (3.8, 3.9)	2.5 (2.3, 2.7)	9.6 (9.5, 9.6)	6.4 (6.3, 6.4)	4.6 (4.5, 4.6)	2.4 (2.4, 2.4)
2013	461	209	252	56.2 (55.6, 56.8)	2.3 (2.2, 2.3)	129.2 (128.9, 129.6)	79.7 (79.4, 80.1)	6.2 (6.2, 6.3)	2.8 (2.7, 2.8)
2014	406	200	206	48.9 (48.4, 49.5)	2.9 (2.8, 3.0)	143.1 (143.0, 143.3)	56.8 (56.7, 57.0)	10.2 (10.1, 10.2)	2.9 (2.9, 2.9)
2015	255	68	187	17.6 (17.2, 18.0)	2.3 (2.2, 2.4)	40.5 (40.4, 40.7)	27.4 (27.3, 27.6)	4.8 (4.7, 4.8)	2.5 (2.5, 2.5)
2016	405	34	371	11.0 (10.9, 11.2)	2.6 (2.5, 2.8)	29.2 (29.2, 29.3)	4.7 (4.7, 4.8)	6.7 (6.6, 6.8)	2.7 (2.7, 2.8)
2017	289	17	272	4.5 (4.3, 4.7)	1.1 (1.0, 1.1)	4.8 (4.7, 4.9)	12.2 (12.1, 12.3)	2.1 (2.1, 2.1)	2.0 (2.0, 2.0)
2018	189	3	186	2 (2.0, 2.0)	1.5 (1.4, 1.5)	2 (2, 2)	1 (1, 1)	3.9 (3.9, 4.0)	3 (3, 3)
**All**	**5272**	**2808**	**2464**	**537 (530.8, 543.1)**	**2.8 (2.7, 2.9)**	**2360.2 (2358.1, 2362.4)**	**447.7 (445.6, 449.9)**	**13.0 (12.1, 13.2)**	**3.1 (3.0, 3.1)**

In order to infer relationships between the cases, the occurrence data is utilized in combination with weather and population mobility data. Daily weather data includes minimum and maximum temperatures for the period of October 2001 to September 2018, recorded at 216 weather stations across Queensland and was obtained from the Australian Bureau of Meteorology. For human mobility data, we constructed yearly origin-destination matrices for the studied period from National Visitor Surveys (NVS), International Visitor Surveys (IVS) and Twitter data. The surveys of national and international visitors were conducted by Tourism Research Australia and record the different SA4 regions that individuals visited. For national visitors the SA4 region of residence is also recorded. NVS data is available between 1998 and 2015, IVS data is available between 2005 and 2015. The Twitter data supplements the IVS and NVS datasets as a source of intra-region mobility. It was collected in 2015 and contains 925,945 trips from 79,271 individuals [27]. For the missing years, we apply an auto-regressive moving average model [28], a common method for forecasting time-series to estimate movement between regions.

### Disease spread network

Utilizing the dengue occurrence data along with the population mobility and meteorology datasets, we construct the most likely disease spread network (DSN) that depicts the spatio-temporal spread of dengue in Queensland. The DSN is a directed acyclic graph, where nodes represent dengue cases and arcs represent probable relationships between them. The construction of the network is a two stage process. In the first stage, we draw arcs by applying the epidemiological soundness criteria and in the second stage, we prune the network using the LIPP model [10] to identify the most likely transmission paths. The epidemiological soundness of an arc depends on the spatio-temporal and clinical characteristics of the cases that are being considered to be connected by the arc. More specifically, in order for two cases *p* and *c* to be connected via an arc (*p* → *c*), they must fulfill the following criteria;

- the exposure period of *c* must overlap with the transmission period of *p*,
- the region of acquisition of *c* must coincide with region of residence of *p* (applied on SA4 level),
- *p* and *c* must not be infected by different serotypes of dengue.

We describe the relationship between *p* and *c* as that of a parent and child, where *p* is the parent case and *c* is the child case. To fulfil the first criterion, we estimate the IIP and average EIP for each case using a Gamma and log-normal time-to-event model respectively, where the latter incorporates temperature sensitivity through a co-variate (see S2 Text for more details) [11]. The LP is estimated by decreasing the estimated IIP value by 2 [19]. The remaining criteria can be tested using the information provided in the dengue occurrence dataset. Since IIP is randomly drawn from a gamma distribution its randomness may impact the structure of the network. To account for this variability in our analysis we repeat the network construction process through Monte Carlo’s simulation and present disagreement of measures across various instances with a 95% confidence interval wherever applicable. The simulation converges when there is 95% probability that the relative error of the sampling distribution of means is at most 1%. The EIP on the other hand, is drawn from a log-normal distribution that generates the mean of all possible EIPs for a given temperature. The mean EIP remains unchanged across independent draws for the same temperature.

At the end of the first stage of network construction we obtain a probable DSN that logically aligns with the epidemiological characteristics of dengue. Yet it is not an acceptable representation of disease spread. In nature, we expect each dengue case to have exactly one parent case. However, the above criteria do not prevent a node from having multiple in-coming arcs, implying a practical impossibility that it has been caused by more than one case. The second stage is required to address this problem.

In the second stage, LIPP model is used to prune the excess arcs. The model estimates pair-wise causation probabilities for dengue cases using a multivariate *Hawkes process* [29]. It models disease spread across a heterogeneous social system by incorporating three major counterbalancing factors: (i) exogenous influence covering environmental heterogeneity, (ii) endogenous influence attributed to macro level interactions between meta-populations, and (iii) a time decay kernel. In this context, the exogenous influence incorporates the importation of the infection from abroad and the availability and density of the vector in the regions (given that dengue is non-endemic in Australia, importation of infections play a crucial role in the local spread of the disease). The endogenous influence captures the impact of inter-region human mobility. An infected individual gradually recovers from the infection through a natural immune response, decreasing their infectivity over time, this effect is integrated by the the time decay kernel. The estimates are computed using the disease occurrence and population mobility data (see S2 Text for more details). The estimates generated by the model are used to break ties between multiple parents of a child resulting in the most likely parent-child arc being preserved.

In the context of the disease spread network, we define a case to be fertile if the node representing the case has at least one out-going arc and infertile otherwise. Similarly the reproduction of a case denotes the number of out-going arcs of the node representing the case.

### Statistical analysis

Utilizing the disease spread network, we enquire whether morbidity is related to delay in disease response. Furthermore, we test varying response delay thresholds for what may represent timely surveillance, and gauge their impact on various parameters associated with the incidence of dengue. In particular, we considered the impact of calibrated disease response on the occurrence and reproduction of the cases as well as the size and depth of the outbreaks. The thresholds are enforced by removing the child cases (and their descendants) whose exposure period most likely occurred after the response efforts have been conducted for the parent case. The cases removed through this process are denoted as *prevented cases*. The removal of these cases effects all four indicators described above. We note that it is not possible to prevent a case with an unknown parent by reducing response time. Similarly, a childless case has zero reproduction, which cannot contract further through reduction in response time. Therefore, we restricted our analysis of case occurrence and reproduction to the cases with a known parent and to the cases that has at least one child, respectively. We also measure the impact of variation in response delay in terms of outbreak size and number of generations. Since an outbreak consisting of a single case cannot be reduced in size by limiting response delay, we restrict our analysis to the outbreaks of size at least 2. Similarly, when considering the reduction in number of generations, we limit our analysis to the outbreaks with at least two generations as the number of generations cannot be reduced for a singleton. All results are reported in comparison to the baseline established in the original data.

We conduct a spatio-temporal analysis of the notification delay components by dividing it into three parts namely, *patient delay*, *laboratory delay* and *clerical delay*. The patient delay is the time elapsed (in days) since the onset of symptoms to the date of specimen collection, which can be interpreted as a combination of the delay on part of a patient before they seek medical attention and the delay in clinical diagnosis by the treating doctor. The time taken by a laboratory from the date of specimen collection to the date of diagnosis is called the laboratory delay. Lastly, the time elapsed between the date of diagnosis and the date of notification is called clerical delay. For further insights we apply the spatial analysis of the lab and patient delays on a finer (locality) level. Additionally, we classify the reported cases by their seasonal and demographic attributes to identify any impact these attributes may have on the lab and patient delays. For seasonality analysis we classify the cases by month of onset while the demographic analysis is conducted through classification into age (9 classes containing patients of age 1 to 90 with each class consisting of ten consecutive discrete values (e.g., 1–10, 11–20)) and gender groups. The outcome of theses analyses can prove useful in providing guidelines on targeted resource allocation for public awareness as well as to improve diagnostic abilities and turn-around times for doctors and laboratories involved.

## Results

### Case fertility and response delay

Of the 5,272 reported cases, 537 (10%) were estimated to be fertile. Moreover, of the 2,808 locally acquired case, a parent was found for 2,360 (84%) while no suitable candidates were found for 448 (16%) (see Table 1 for details on fertility and parent-child relationship estimates). The years with the highest number of fertile cases mostly correspond to years with most number of reported cases. However, in terms of proportion of reported cases, 2005 was estimated to have the highest proportion of fertile cases (18%) followed by 2006 and 2004 with 17% fertile cases each. In terms of region-wise distribution, Cairns was estimated to have the highest number of fertile cases (350) followed by Townsville (112) and Queensland-Outback (44). Proportionally, Townsville led Cairns with 22% fertile cases compared to 15%, with Queensland-Outback (10%) at the third spot.

We observed a positive relation between the number of fertile cases and the length of transmission period. A longer transmission period allows time for the infected mosquitoes to bite more people, which leads to a higher chance of fertility. We note that the transmission period can be cut-short through the deployment of disease response. Therefore, a longer transmission period is a consequence of delayed response. Our estimation showed that the fertile cases had a mean transmission period of 10 days. Moreover, the first, second (median) and third quartile transmission periods were observed to be of 5, 8 and 13 days, respectively. On the other hand, only 5% fertile cases had a transmission period of 2 days or less.

**Fig 2:**
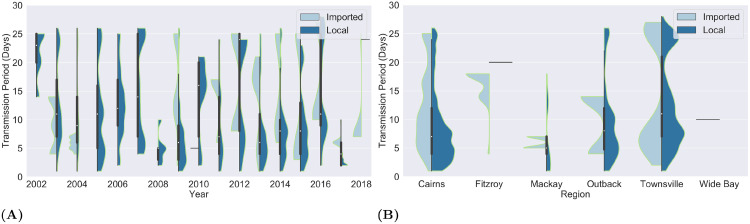
Transmission periods of fertile cases. The violin charts in (A) and (B) depict the length of transmission periods in fertile cases by year and region (SA4) of case occurrence. Each element of the violin chart contains a box and whiskers plot sandwiched by the distribution curves of the transmission periods of imported and locally acquired fertile cases.

A year-wise breakdown of the fertile cases (see Fig 2A) presented a similar pattern. With the exception of 2017(4 days), overall the fertile cases reported each year had an estimated median transmission period of at least 5 days (IQR = 5–13). Spatially (Fig 2B), fertile cases in Townsville were estimated to have the longest transmission periods with the mean, first, second and third quartile of 13, 7, 11 and 21 days, respectively. Overall, the median transmission period exceeded 5 days across all regions. For Cairns and Queensland-Outback in particular, we observed a median transmission period of at least 7 and 8 days, respectively. The distributions of the length of transmission periods of imported and locally acquired fertile cases show that the descriptive statistics are not skewed by a particular group or a small number of very long transmission periods.

### Temporal calibration of response

**Fig 3:**
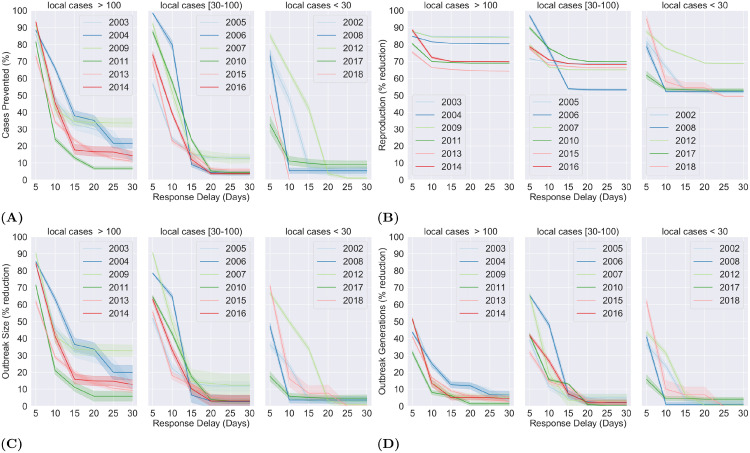
Temporal calibration of disease response. The impact of varying response delay thresholds on the dynamics of dengue occurrence. (A) plots percentage of cases prevented while (B), (C) and (D) plot percentage reduction in reproduction, outbreak size and number of generations against varying response delay thresholds. The reproduction denotes the number of secondary cases per fertile case, the size and generations of an outbreak denotes the number of cases and number of generations in the infection chain of the outbreak, respectively.

We tested 6 response delay thresholds between 5 and 30 days (inclusive) with an interval of 5 days. In general, the highest reduction was noticed across all parameters for the response delay threshold of 5 days. Another commonality was the large difference between the response times of 5 and 10 days (see Fig 3). Particularly, there was an average reduction of 87% [95% CI, (86, 88)] and 83% [95% CI, (83, 84)] in case occurrence and reproduction respectively, when a response time of at most 5 days was enforced. A sharp decline was observed in the number of prevented cases, which contracted by 41% (to 46% [95% CI, (43, 48)]) and a further 15% (to 31% [95% CI, (28, 33)]) when the response delay was extended to 10 and 15 days. The difference was not as significant for higher response delay thresholds.

The average reduction in case reproduction also declined with increasing response time, though not as dramatically, ranging between 71% [95% CI, (71, 71)] and 83% [95% CI, (83, 84)]. In terms of year-wise classification (Fig 3A), the years that recorded more than 100 locally acquired cases(2003, 2004, 2009, 2011, 2013 and 2014) saw a significant reduction in incidence across all response delay thresholds. On the other hand, for the years with less than 100 locally acquired cases, the proportion of prevented cases fell to less than 20% when the response delay was set to 15 days and above. The analysis of the annual breakdown of the case reproduction (Fig 3B) showed that the overall averages were skewed by the 3 years with the highest local incidence where the reduction consistently remained between 80% and 90% across all 6 response thresholds. However, this was not the case for the years with lower occurrence where in most cases the reproduction declined between 30% to 50% when the response time was extended from 5 to 10 days.

The outbreak size followed a trend that was overall similar to that of prevented cases. An 80% [95% CI, (79, 81)] reduction was estimated in average outbreak size when the response delay was fixed at 5 days. Once again, a sharp decline (38%) was observed when the response delay was extended to 10 days with outbreaks shrinking by 42% [95% CI, (39, 45)] on average. It shrunk by another 18% (to 24% [95% CI, (21, 28)]) before flattening, when a response delay of at most 15 days was applied. The year-wise analysis of the outbreak sizes (Fig 3C) revealed trends that were quite similar to that of prevented cases. The years with less than 100 locally acquired cases showed little reduction at response thresholds of 15 days and above, while the outbreaks in years with the highest occurrence shrunk significantly at both small and large response delay thresholds. The number of generations was the least affected parameter in our analysis, with a 47% [95% CI, (45, 48)] average reduction for a response delay threshold of at most 5 days. It dropped swiftly to 19% [95% CI, (16, 21)] and then 7% [95% CI, (5, 9)] when the response delay was extended to 10 and 15 days before somewhat flattening to under 5% at the response delay of at most 25 and 30 days. Analysis of the year-wise classification (see Fig 3D) showed that a response delay of 5 days resulted in a 30 to 50% contraction in the number of generations for most years with the smallest reduction noted at 18%. This fell to 10% and below for most years when the response was delayed by 15 days. Furthermore, little to no reduction was noted at the response thresholds of 25 and 30 days.

### Reducing response delay

**Fig 4:**
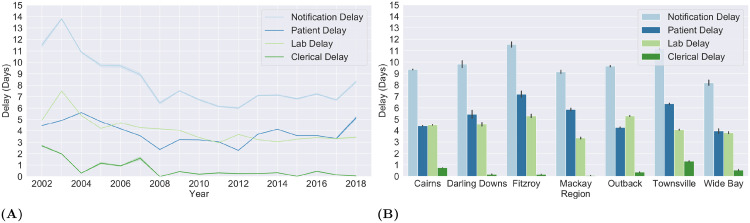
Notification delay and its components. A temporal (A) and spatial (B) depiction of notification delay and its components for the dengue cases reported in the vector present regions of Queensland, Australia. The cases are grouped by the year and region (SA4) of occurrence, respectively. The patient delay is the time elapsed (in days) since the onset of symptoms to the date of specimen collection. The time taken by a laboratory from the date of specimen collection to the date of diagnosis is called the laboratory delay. And, the time elapsed between the date of diagnosis and the date of notification is called clerical delay.

A temporal plot of the notification delay and its components is provided in Fig 4A. The patient delay showed no clear temporal pattern. Though a couple of dips below three days in the years 2008 and 2012 coincided with a drop in case fertility to under 5%. This pattern was not noticed for other year (2002, 2016, 2017 and 2018) with under 5% fertile cases. The lab and clerical delays gradually declined as the years progressed. The average lab delay decreased from nearly 7.5 days to just over 3 days between 2003 and 2018 with minor hikes in 2006 and 2012. Similarly, the average clerical delay mostly fluctuated between 1 and 3 days for years between 2002 and 2007 (except 2004), fell to 0 in 2008 and consistently remained under 0.5 days for the following years. Overall, the variation in notification delay from 2008 onward were largely influenced by lab and patient delays as the clerical delays flattened.

**Fig 5:**
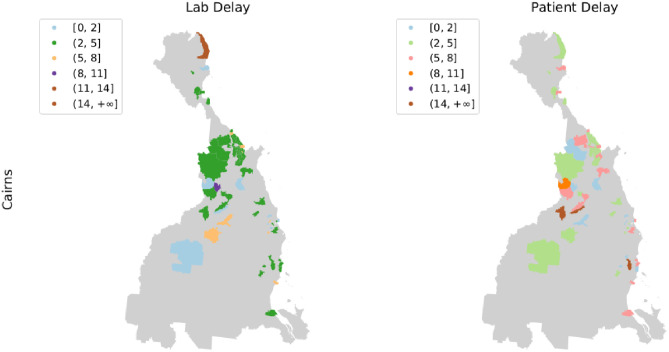
Spatial analysis of the notification delay components. Geo-spatial depiction of the average lab and patient delays in the localities of Cairns.

Spatially (Fig 4B), an average patient delay of 4 days or above was observed for all regions with Townsville and Fitzroy reporting an average delay of 5 and 6 days respectively. Similarly, An average lab delay of at least 4 days was reported for all regions, except Mackay. Townsville saw the longest clerical delay (1.1 days) on average across all regions followed by Cairns (0.9 days), Wide Bay (0.6 days) and Queensland—Outback (0.4 days). Overall, an average notification delay of at least 9 days was noted in all regions except Wide Bay (8.1 days). Of the 3 regions with the highest incidence (Cairns, Queensland—Outback and Townsville), Townsville encountered the longest notification delay (11.1 days) on average. For a finer spatial analysis, a geo-spatial depiction the boundaries for Cairns and its localities are rendered using shape files obtained from Australian Bureau of Statistics [30] that are publicly available under the Creative Commons licence of the average lab and patient delay in the localities of Cairns is provided in Fig 5 (see S1 Fig, for similar results on other vector present regions of Queensland).

**Fig 6:**
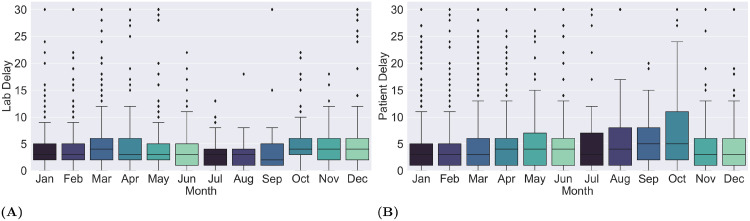
Impact of seasonality on lab and patient delays. The box and whisker plots depict monthly statistics of the lab (A) and patient (B) delays where cases are grouped by month of occurrence.

To analyze the seasonal and demographic trends in patient and lab delays, we classified the reported cases by their month of occurrence, age and gender. We noticed that the longest patient delays on average were recorded during the spring season between August and October (see Fig 6B). A regional (SA4 level) breakdown of this analysis is presented in S3 Fig. On the other hand, the lab delays peaked during the fourth quarter with the median delay of 4 days, while the shortest delays occurred in the third quarter (Fig 6A). Applying seasonal analysis on SA4 level (see S2 Fig), we noticed similar trends in Cairns and Townsville. In Cairns, the longest lab delays on average were recorded during the months of April (6 days), October and December (4 days). In Townsville, the average delays peaked during September and October (5 and 6 days, respectively). The remaining regions showed no clear pattern. Lastly, in order to identify demographic groups of patient for targeted awareness campaigns, we performed an analysis of the patient delay trends through classification of the reported cases by age and gender (S4 Fig). We found that in male patients, most cases with a patient delay of 4 to 5 days were observed in the age group 30 to 35 years. Moreover, the largest number of cases with a delay of 10 or more days were observed in patients of age 45 to 60. On the other hand, a delay of 4 to 5 days was most frequently observed in female patient of age groups 20–35 and 45–50 years, the same groups also had the highest frequency of cases with delay of 10 and more days.

## Discussion

We apply the epidemiological soundness criteria to construct a disease spread network. The construction of the network implicitly quantifies the fertility and reproduction of the cases as well as the size and depth of the outbreaks, all important parameters for an epidemiological analysis. Although our response delay threshold analysis was focused on dengue occurrence in Australia, it can be applied to other infectious diseases (particularly vector-borne diseases) and/or countries where the data and the epidemiological characteristics of the diseases render it feasible.

We empirically confirmed that most fertile cases had a long response delay. However, we also found that a long response delay did not necessarily lead to a case being fertile. There are a range of factors that may prevent the vector from feeding on an infectious individual despite a long transmission period, including housing with air conditioning and fly nets, the use of mosquito repellent and confinement to a mosquito free space (e.g., hospital) during illness. Moreover, vector presence data is collected through sampling (by setting up mosquito traps at various locations in a region). While this may be the only viable option, it is insufficient to confirm the presence of the vector in all parts of a region. The involvement of these factors and a lack of data on them prevent us from analysing the relationship between response delay and infertile cases.

Most disease surveillance systems are affected by a degree of under-ascertainment leading to uncertainty on the actual incidence of the disease. Our analysis highlighted the under-ascertainment of dengue occurrence in Queensland. Given that dengue is non-endemic in Australia, each outbreak is expected to be rooted in an overseas acquired case. However, while constructing the disease spread network, we were unable to identify a parent case for approximately 16% of the locally acquired cases. Furthermore, the years 2002 and 2005 to 2008 were estimated to have no imported fertile cases despite having local transmission. These findings are an indication of the under-ascertainment of dengue occurrence in Australia.

A short EIP and delayed response have been previously attributed to be the major contributing factors for large dengue outbreaks in 2009 [31]. Our analysis confirmed this and revealed that it was also the case for other years of large dengue outbreaks including 2003, 2004 and 2014, by noticing significant reduction in average occurrence, reproduction and outbreak sizeacross all response delay thresholds.

A sharp decline was observed in reduction of outbreak generations at the response thresholds of 10 and 15 days indicating that most outbreaks had a short generation interval (the difference between the infection time of a primary case and one of its secondary cases). This observation is a possible explanation for a similar fall in the reduction of occurrence and outbreak size as the response efforts may be committed when the first few (one and possibly more) generations of cases have already produced child cases.

The Queensland Health Dengue Management Plan states that the treating doctors are required to report any suspicious cases of dengue before the laboratory confirmed diagnosis. However, over the 16 years of dengue occurrence covered in this study we found only 34 (0.6%) cases that were notified before a laboratory diagnosis, stressing the need for a wider awareness on the importance of reporting suspicious cases and better understanding of the dengue symptoms for clinical diagnosis.

We observed that the number of locally acquired cases in Cairns and Townsville regions remained consistently low over the last few years of the study (14 and 11 cases respectively since 2016) despite longer than ideal response delays. We note that this unusual trend can be attributed to the deployment of the Wolbachia (a natural bacteria that reduces the mosquitoes’ ability to transmit infections like dengue, Zika and chikungunya) infected mosquitoes in these regions since 2011 [32].

The seasonality analysis of the notification delay components showed that the shortest and the longest lab delays (on average) coincided with the off-peak and peak season of dengue occurrence in Queensland, respectively. One interpretation of this trend is that the performance of the labs may have suffered under higher case load which may be due to limited testing capacity. However, this interpretation is countered, to an extent, by a decline in lab delays during January and February, the months that make up the later part of the peak season in Queensland. A similar pattern emerged in the region-wise seasonality analysis where lab delays peaked during fall (April) and spring (October) season. Interestingly, April and October are also the months of school holidays in Queensland, which leads to the possibility that the longer lab delays may have been due to the diminished capacity of the labs with staff taking time off work. For patient delays, we observed a jump during the spring season between August and October. Since the spring season is high time for hay fever in Queensland, which presents symptoms that are similar to that of dengue, the rise in patient delays may be due to initial clinical/personal misdiagnosis of the dengue cases.

A comparison with the existing literature is important to justify the motivation for a new approach. We refer to Swaan et al. [12] for a comprehensive systematic review of the literature on timeliness of the notification systems for infectious diseases. The review included 48 studies from 17 countries published over almost two decades (2000 to 2017). A majority of the studies included in the review measure the timeliness of the notification system by comparing the case notification delays to predefined (39), standardized (45) and/or disease specific timeframes (8). These timeframes were defined through legislation, local rules or by the authors. In some cases the timeframes were obtained by estimating the average number of hypothetical consequent cases from the average length of infectious period [33]. Our methodology, on the other hand, identifies these timeframes by enforcing various response delay thresholds and measuring their impact on the actual disease occurrence. The results were often reported as the proportions of the cases with notification delay falling on either side of the timeframes. In some cases (13 studies) a comparison was provided between the timeliness of the electronic and conventional system. In most cases the notification delay was divided into components with descriptive statistics provided for each component. Similar pattern was observed in more recent studies as well [14–18]. Instead of reporting the proportion of cases that meet and/or does not meet a specific time frame, we rather report the impact of notification delay on the parameters that define the occurrence of the disease. Given the fundamental difference in the modes of measurement, a fair quantitative comparison is not feasible.

Like any data driven model, the accuracy of our results is subject to the quality of the underlying data. We understand that the dataset we have used may introduce some bias. For instance, there may be a degree of under-ascertainment in dengue cases which cannot be captured by the dengue occurrence data. Similarly, the NVS, IVS and Twitter data may not be an exact representation of population mobility due to survey methods, willingness for participation as well as the availability of Internet and penetration of social media in the underlying population.

We acknowledge that our specification of the spatial and temporal conditions for the parent-child relationships may introduce some bias. Recall that we require that the place of acquisition of the child case must match the place of residence of the parent. Even though the occurrence data provide the region of acquisition for each case, there is still a possibility that a parent may have caused a child by traveling (outside its place of residence), while infectious, to the region of acquisition of the child case. Nevertheless, in our setting, low inter-region mobility and incidence rate (per region) indicate that such instances would have been a rare occurrence. On the temporal scale, we require the transmission period of a parent to overlap with the time of exposure of a child. Since the transmission period and time of exposure are defined through the estimated values of IIP and EIP (which are drawn from time-to-event models), our parent-child relationships may (in some cases) not represent the real-world causation-relations accurately. Although we attempt to account for these variations by applying the Monte Carlo method for the construction of the disease spread network and using the time-to-event models that were fitted to experimental data, it is hard to verify the accuracy of our estimates in the absence of actual case-wise data on the incubation periods.

We assume that the case transmission period occurs continuously between its lower (earliest concluding EIP) and upper (latest concluding vector transmission period) bounds. Nevertheless, in real world the period may occur in intermittently (between the bounds). This overestimation may lead to invalid parent-child relationships in two ways. One, we may attribute a child case to multiple parents where only one of them is its true parent. Two, we may attribute a child case to one or more cases where none of them is its true parent. We resolve the first instance by identifying the most likely parent of the case by applying the LIPP model [10]. However, the second instance most likely occurs due to the under-ascertainment of the disease and is not addressed here due to lack of relevant data.

Our work points to various future research directions. A natural extension of the work would be to apply this methodology to similar datasets collected in different geographical (beyond Australia) and epidemiological (other infectious diseases e.g., Zika) settings. Among other directions, one interesting extension of the study would be the development of a case ranking system for laboratories, where cases would be ranked by a risk function. The risk function could be based on various parameters including time since onset, place of residence and weather conditions at the place of residence since onset. Another interesting problem to pursue would be to estimate the economic burden of ineffective surveillance. It could cover among other costs, the spending on providing healthcare for larger number of patients as the outbreaks grow and the economic losses due to days spent off work. Similarly, an evaluation of the economic costs required for realisation of the identified disease response thresholds would be equally interesting.

## Conclusion

We construct a disease spread network by applying the epidemiological soundness criteria in combination with the LIPP and time-to-event models to the disease occurrence, meteorological and human population mobility data. Using this network, we empirically confirm that high morbidity relates positively with delay in disease response. Moreover, we identify what constitutes timely surveillance by applying various disease response delay thresholds to the network and report their impact on case fertility, reproduction, outbreak size and generations. Lastly, we identify the components of the disease surveillance system that can be calibrated to achieve the identified thresholds. Our methodology can be utilized to provide guidelines on spatially and demographically targeted resource allocation for public awareness campaigns as well as to improve diagnostic abilities and turn-around times for the doctors and laboratories wherever required. The surveillance thresholds obtained in this study can be utilized to define a case ranking system for the pathology labs that can help prioritize testing of the cases that are more likely to lead to a larger outbreak.

## Supporting information

S1 Fig Finer spatial analysis of lab and patient delays. (PDF)

S2 Fig Seasonality analysis of the lab delays by region. (PDF)

S3 Fig Seasonality analysis of the patient delays by region. (PDF)

S4 Fig Demographic analysis of the patient delays considering the age and gender of the patients at the time of disease onset. (PDF)

S1 Text Case transmission period. (PDF)

S2 Text LIPP and time-to-event models. (PDF)
