# Supplementary material for: Timely surveillance and temporal calibration of disease response against human infectious diseases
Source: PLoS One. 2021 Oct 18;16(10):e0258332. doi: 10.1371/journal.pone.0258332 (PMC8523075; doi:10.1371/journal.pone.0258332)
Supplement: S1 Fig — (PDF) [file pone.0258332.s001.pdf]

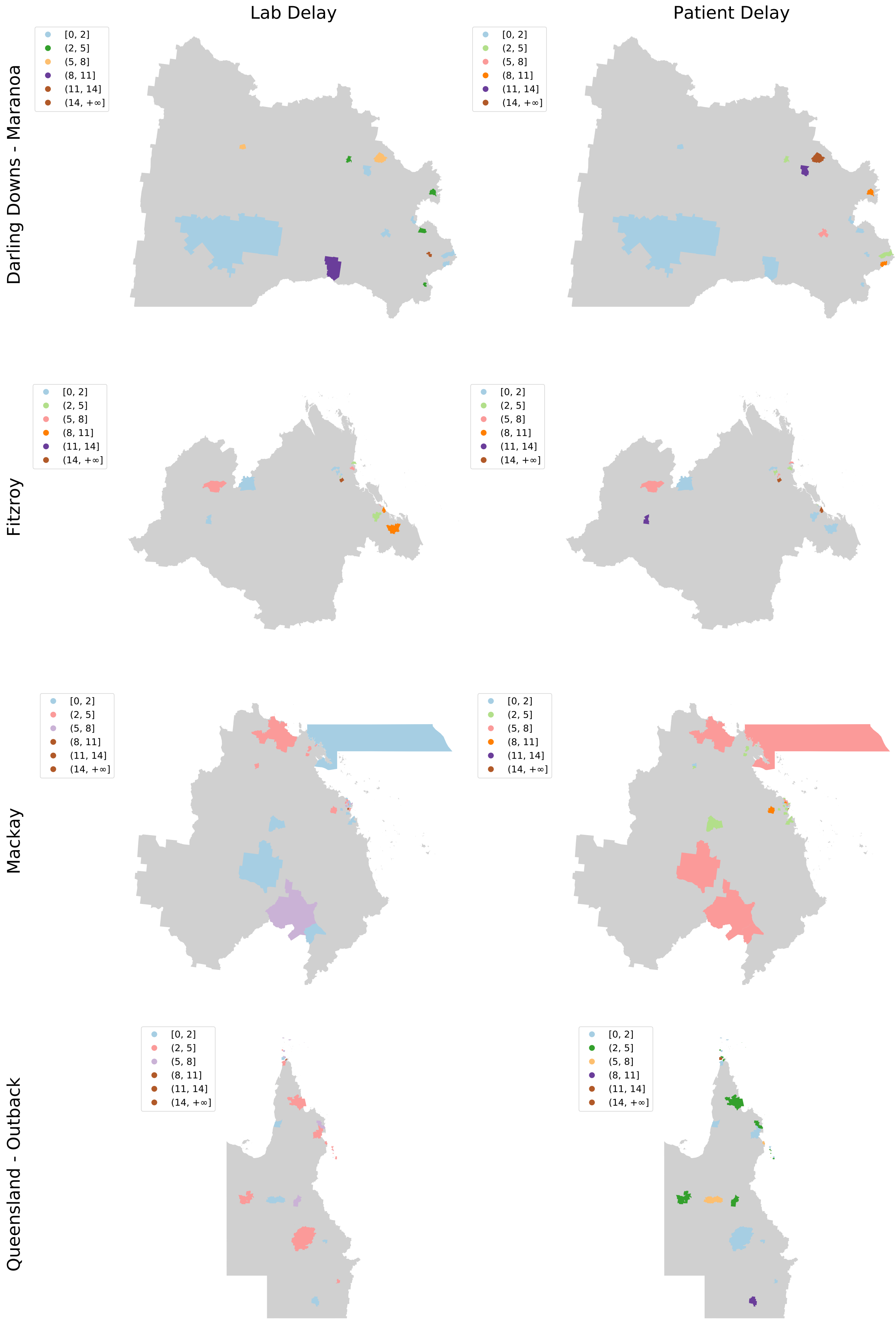

Lab Delay
Patient Delay
Darling Downs - Maranoa
[0, 2]
(2, 5]
(5, 8]
(8, 11]
(11, 14]
(14, +∞]
[0, 2]
(2, 5]
(5, 8]
(8, 11]
(11, 14]
(14, +∞]
Fitzroy
[0, 2]
(2, 5]
(5, 8]
(8, 11]
(11, 14]
(14, +∞]
[0, 2]
(2, 5]
(5, 8]
(8, 11]
(11, 14]
(14, +∞]
Mackay
[0, 2]
(2, 5]
(5, 8]
(8, 11]
(11, 14]
(14, +∞]
[0, 2]
(2, 5]
(5, 8]
(8, 11]
(11, 14]
(14, +∞]
Queensland - Outback
[0, 2]
(2, 5]
(5, 8]
(8, 11]
(11, 14]
(14, +∞]
[0, 2]
(2, 5]
(5, 8]
(8, 11]
(11, 14]
(14, +∞]

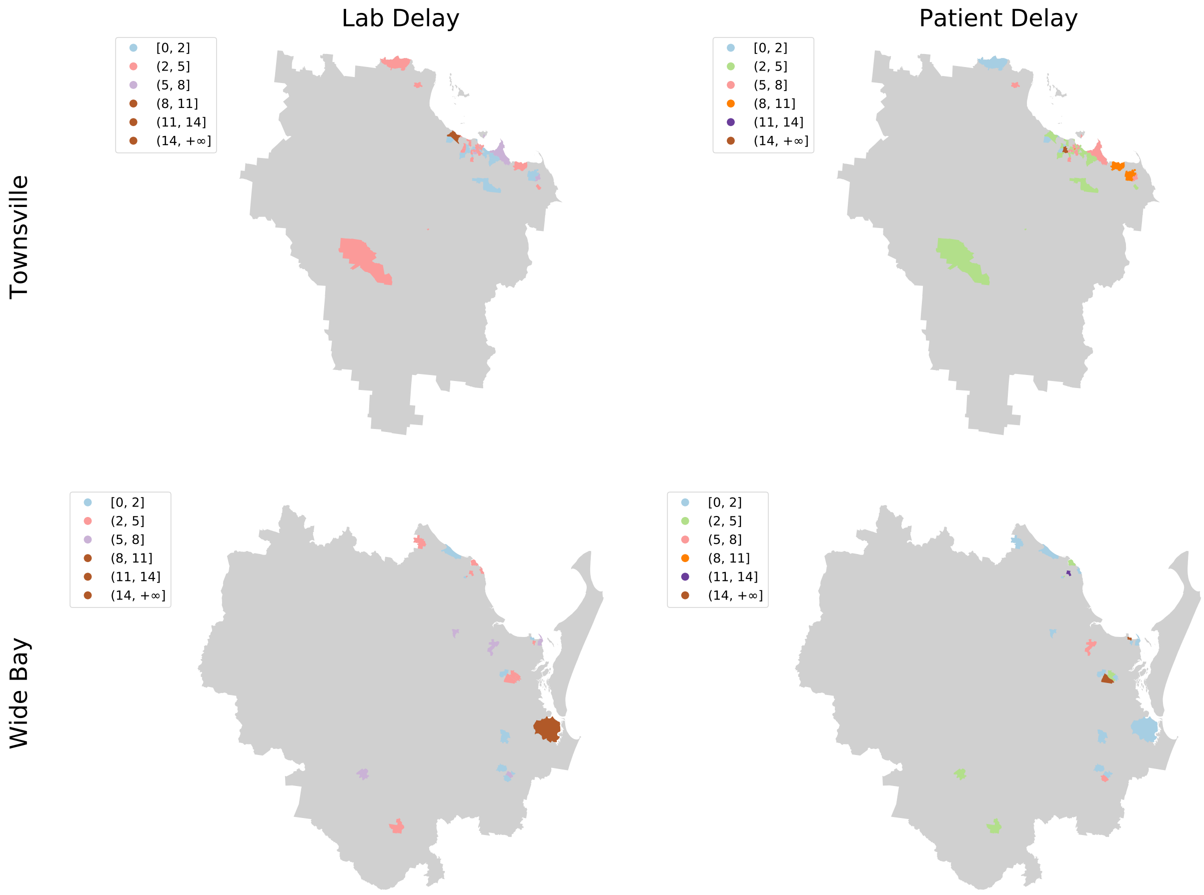

Fig S 1. Finer Spatial Analysis of Lab and Patient Delays. Geo-spatial depiction of the average lab and patient delays in the localities of the vector present regions of Queensland, Australia (excluding Cairns). In each map the localities are colored to represent the average delays in days. The legend attached to each map maps the colors to the corresponding values.

Disclaimer: The boundaries for SA4 regions and their localities are rendered using shape files obtained from Australian Bureau of Statistics [1] that are publicly available under the Creative Commons licence.

References

[1] Australian Bureau of Statistics. Australian Statistical Geography Standard (ASGS): Volume 1 - Main Structure and Greater Capital City Statistical Areas; 2016. [Online]. Accessed on December 12, 2020 from: https://www.abs.gov.au/ausstats/abs@.nsf/mf/1270.0.55.001, under Creative Commons licence: https://creativecommons.org/licenses/by/2.5/au/legalcode.
