# Supplementary material for: Timely surveillance and temporal calibration of disease response against human infectious diseases
Source: PLoS One. 2021 Oct 18;16(10):e0258332. doi: 10.1371/journal.pone.0258332 (PMC8523075; doi:10.1371/journal.pone.0258332)
Supplement: S3 Fig — (PDF) [file pone.0258332.s003.pdf]

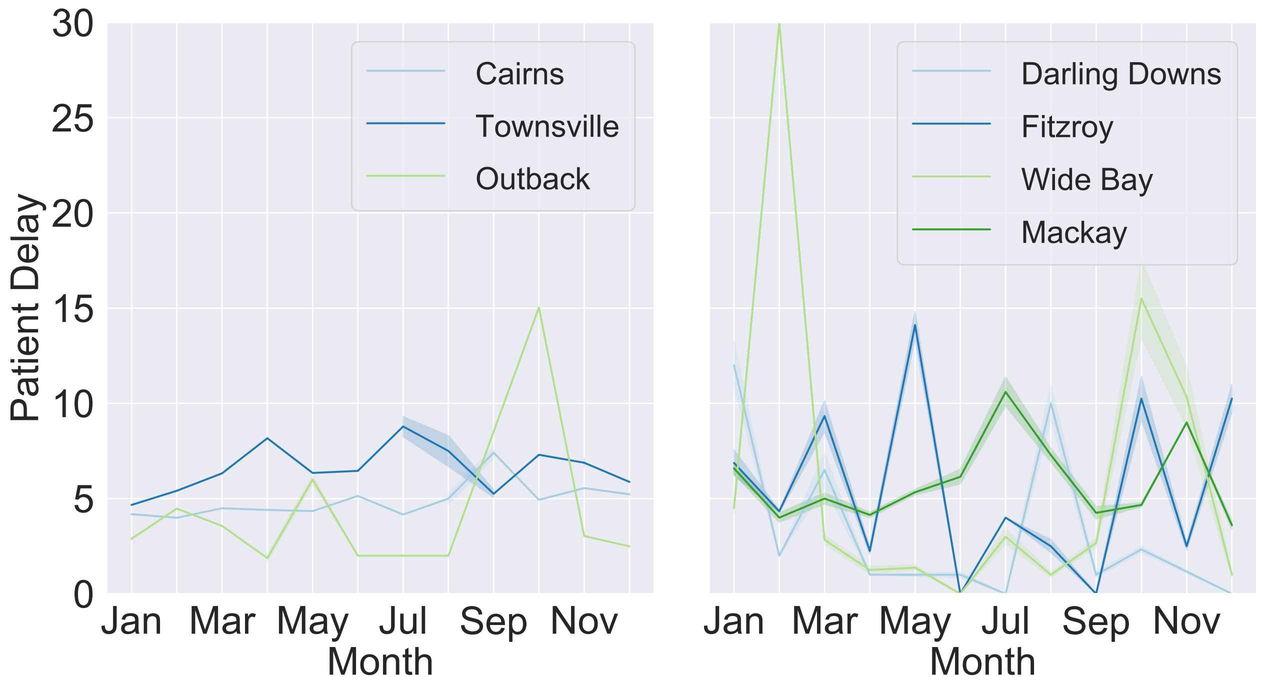

Fig S 3. Seasonality analysis of the patient delays. The line chart depict average patient delays by SA4 regions. The chart is split into two parts, for the sake of visibility, separating the regions with low occurrence (< 400 cases) from that of high occurrence of dengue cases (> 400 cases).
