# Supplementary material for: Timely surveillance and temporal calibration of disease response against human infectious diseases
Source: PLoS One. 2021 Oct 18;16(10):e0258332. doi: 10.1371/journal.pone.0258332 (PMC8523075; doi:10.1371/journal.pone.0258332)
Supplement: S4 Fig — (PDF) [file pone.0258332.s004.pdf]

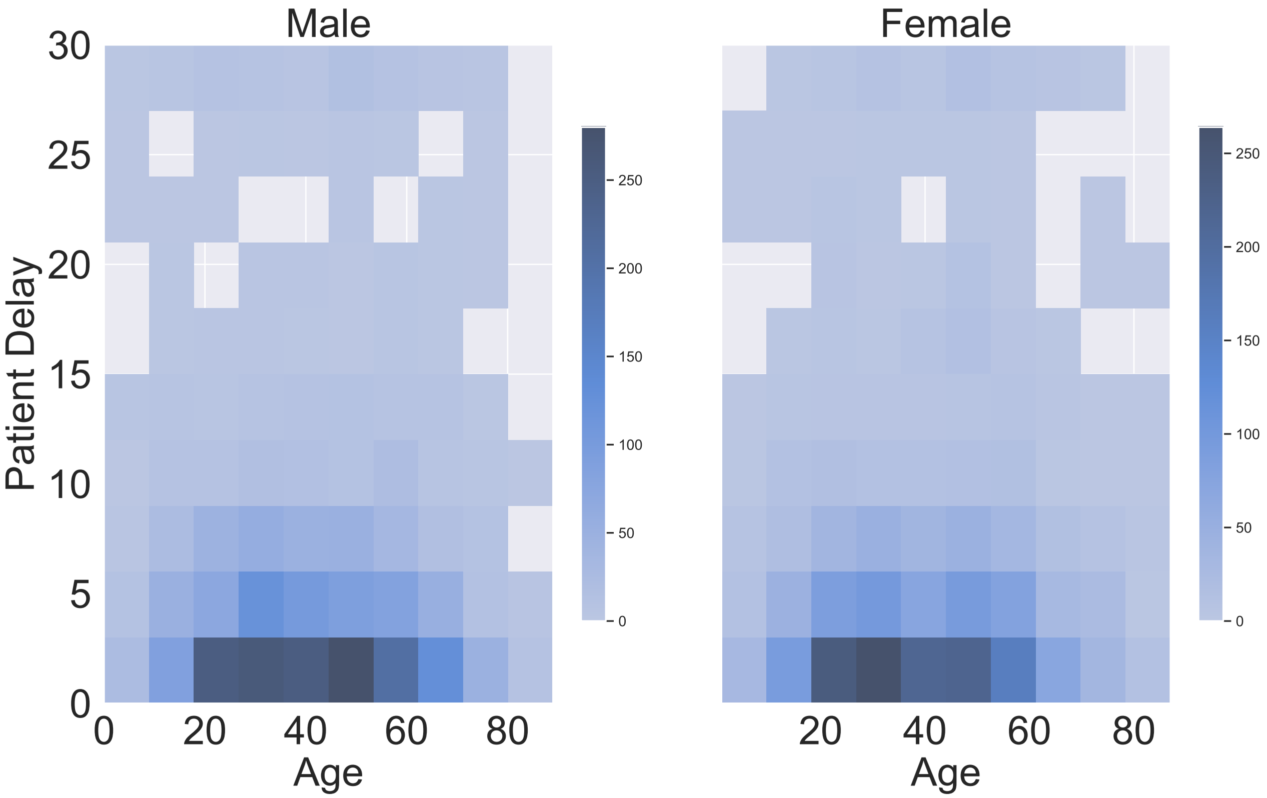

Fig S 3. Demographic analysis of the patient delays. The bivariate histograms show the distributions of the patient delays by age of the patient at the time of disease onset. The histograms are divided into groups by patient gender.
