# Supplementary material for: Timely surveillance and temporal calibration of disease response against human infectious diseases
Source: PLoS One. 2021 Oct 18;16(10):e0258332. doi: 10.1371/journal.pone.0258332 (PMC8523075; doi:10.1371/journal.pone.0258332)
Supplement: S1 Text — (PDF) [file pone.0258332.s005.pdf]

Timely Surveillance and Temporal Calibration of Disease Response against Human Infectious Diseases

S1 Text. Case Transmission Period

We define a transmission period for each case over which the mosquitoes infected by the case can in turn infect the susceptible population. The length of the transmission period is computed by defining its temporal lower and upper bounds. Observe that a mosquito may bite a person on any day of their infectious period. Since the weather conditions may vary across the days of the period, the length of the EIP (which is heavily influenced by the ambient temperature) of a mosquito biting the person on one day may be different to the one that bites them on another. Therefore, we compute the average EIP for each day of the infectious period and set the earliest concluding EIP (closest to the beginning of the infectious period, on time scale) as the lower bound of the case transmission period. The upper bound occurs at the end of the latest concluding vector transmission period, which either lasts until the date of the disease response actions (up to two days after the case notification) or an average adult mosquito lifespan appended at the end of the latest concluding EIP, whichever occurs first. Any case acquired between these bounds is considered a child case, given that all other soundness criteria are met.
