# Supplementary material for: Timely surveillance and temporal calibration of disease response against human infectious diseases
Source: PLoS One. 2021 Oct 18;16(10):e0258332. doi: 10.1371/journal.pone.0258332 (PMC8523075; doi:10.1371/journal.pone.0258332)
Supplement: S2 Text — (PDF) [file pone.0258332.s006.pdf]

Timely Surveillance and Temporal Calibration of Disease Response against Human Infectious Diseases

S2 Text. Latent Influence Point Process and Time-to-Event Models

Latent Influence Point Process (LIPP)

LIPP [1] is a multivariate Hawkes process [2] that infer transmission routes and infection flow between regions by incorporating three major counterbalancing factors: (i) exogenous influence covering environmental heterogeneity, (ii) endogenous influence attributed to macro level interactions between meta-populations, and (iii) a decay effect. A univariate Hawkes process is given by,

$$\lambda(t) = \mu + \sum_{t_i \leq t} g(t - t_i) \tag{1}$$

where μ denotes the background intensity by an external influence and the second term denotes the endogenous feedback that weighs the influences of past events on future events. In LIPP the background intensity and endogenous feedback are defined by the *exogeneity* and *endogeneity* of a region, respectively. The exogeneity and endogeneity of a region r at time t are given by the Equations 2 and 3.

$$\lambda_r^0(t) = \eta_r \rho_r^0 \tag{2}$$

$$\lambda_r^k(t) = \sum_{t_i \leq t} \zeta(r,k)\xi_k \rho_r^k \phi_r(t - t_i) \tag{3}$$

In Equation 2, η_r represents the environmental heterogeneity (e.g., mosquito presence and density, housing with and without fly-nets and air-conditioning) of r and ρ_r^0 represents the probability of an infectious person arriving in r from abroad. In Equation 2, the function $\zeta_r(r,k)$ returns 1 when r is a regions with vector presence and 0 otherwise. The parameter ξ_k, ρ_r^k and ϕ_r denote the infectiousness (availability of infectious persons and mosquitoes at a given time) of k, the strength of connectivity between k and r with $\sum_{r \in R} \rho_r^k = 1$ (where R is the set of all regions) and the aging effect of the infectiousness of a region. The aging effect is defined by an exponential memory kernel $\phi_r(t - t_i) = exp(-\varphi_r(t - t_i)$, where φ_r is time decay parameter for region r that measure the level of infectiousness decay in r. A multivariate Hawkes process (Equation 5) is obtained through the superposition of the processes defined in Equations 2 and 3. Note that the endogeneity is computed while considering that the population may be divided into more than one meta-populations ($k \in R$ with $|R| > 1$).

$$\lambda_r(t) = \lambda_r^0 + \sum_{k \leq R} \lambda_r^k(t) \tag{4}$$

where $\lambda_r(t)$ denotes the rate of infection in r at time t. In order to compute $\lambda_r(t)$, Bayesian inference was applied to infer the infectiousness ξ_k of each region $k \in R$ while the environmental heterogeneity η_r and the time decay parameter φ_r were inferred using a stochastic expectation-maximization (EM) algorithm. The dengue occurrence and human mobility data was utilised in both methods.

In order to define causal links between the cases a set of latent index variables $Z = \{z_i\}_{i=1}^N$ is defined, where N is the total number of cases. Each latent variable $z_i = \{z_{ij}\}_{j=0}^{i-1}$ is presented by a binary vector of size i containing zeros except for the index of the parent case of the case i (e.g., $z_i = [0, 0, 1, \ldots, 0]$ when ith case is caused by the second case). Note that the zeroth index is designated for an imported case. A Bernoulli distribution prior was imposed on latent index variables. The probabilities of the parent-child relationships between each case n and the cases that has occurred before n are given by

$$p(\{D^{rk}\}_{r=0}^R | \{Z^{rk}\}_{r=0}^R, \xi_k) = (\xi_k)^{\sum_{r=1}^R N^{rk}} e^{-\xi_k \beta_k} \prod_{r=1}^R \prod_{n=1}^{N^{rk}} \zeta(r,k)\rho_r^k exp(-\varphi_r(t_n^{rk} - t(z_n^{rk})) \tag{5}$$

where $\beta_k = \sum_{n=1}^{N^k} \sum_{r=1}^R \frac{1}{\varphi_r} \zeta(r,k)\rho_r^k(1 - exp(-\varphi_r(T - t_n^k)))$ with T being the observation period. D^{rk} denotes the ordered set of cases that occurred in r with parents in k. N^{rk} and Z^{rk} denote the number and latent variables of cases in D^{rk}. Lastly, t_n^{rk} and $t(z_n^{rk})$ present the timestamps of the nth case and its parent in D^{rk}. The above calculation (Equation 5) is performed for each $k \in R$.

Time-to-Event Models

We utilise the censured Bayesian time-to-event models, provided in [3], to estimate the extrinsic and intrinsic incubation periods. Using Markov Chain Monte Carlo methods, Chan et al. [3] fitted four time-to-event models including exponential, Weibull, gamma and log-normal to 146 EIP observations from 8 studies and 204 IIP observations from 35 studies. For each model, they assumed multiplicative hazards using linear covariates, given by $\beta X = \beta_0 + \beta_T T + z_i$, where β_T and z gauge the temperature sensitivity (for EIP) and inter-study random effect, respectively. The quality of the fit was measured by the deviance information criterion (DIC) [4]. They found that the log-normal and gamma models with the probability density functions give by Equations 6 and 7, were the best fit for EIP and IIP, respectively.

$$e^{-\tau(\ln t-\mu)^2/2}t^{-1}\sqrt{\tau/2\pi} \tag{6}$$

$$\lambda^{\nu}t^{\nu-1}e^{-\lambda t}(\Gamma(\nu))^{-1} \tag{7}$$

Note that μ, τ, λ and ν denote the mean, precision, rate and shape parameters respectively, where $\mu(X) = e^{\beta X}$ and $\lambda(X) = \nu/e^{\beta X}$. We use the mean parameter estimates provided in [3] ($\beta_0 = 2.9$, $\beta_T = -0.08$ and $\tau = 4.9$) along with the mean daily temperature to estimate the average EIP value. Similarly, using their parameter estimates, we draw IIP from a gamma distribution of shape 16 and rate 2.7.

References

[1] Kim M, Paini D, Jurdak R. Modeling stochastic processes in disease spread across a heterogeneous social system. Proceedings of the National Academy of Sciences. 2019;116(2):401–406.

[2] Hawkes AG. Spectra of some self-exciting and mutually exciting point processes. Biometrika. 1971;58(1):83–90.

[3] Chan M, Johansson MA. The Incubation Periods of Dengue Viruses. PLOS ONE. 2012;7(11):1–7. doi:10.1371/journal.pone.0050972.

[4] Spiegelhalter DJ, Best NG, Carlin BP, Van Der Linde A. Bayesian measures of model complexity and fit. Journal of the royal statistical society: Series b (statistical methodology). 2002;64(4):583–639.
